# Metabolic Adaptation of Human CD4^+^ and CD8^+^ T-Cells to T-Cell Receptor-Mediated Stimulation

**DOI:** 10.3389/fimmu.2017.01516

**Published:** 2017-11-09

**Authors:** Nicholas Jones, James G. Cronin, Garry Dolton, Silvia Panetti, Andrea J. Schauenburg, Sarah A. E. Galloway, Andrew K. Sewell, David K. Cole, Catherine A. Thornton, Nigel J. Francis

**Affiliations:** ^1^Institute of Life Science, Medical School, Swansea University, Swansea, United Kingdom; ^2^Cardiff University School of Medicine, Cardiff, United Kingdom

**Keywords:** T-cell, immunometabolism, metabolism, T-cell receptor, glycolysis, GLUT1

## Abstract

Linking immunometabolic adaptation to T-cell function provides insight for the development of new therapeutic approaches in multiple disease settings. T-cell activation and downstream effector functions of CD4^+^ and CD8^+^ T-cells are controlled by the strength of interaction between the T-cell receptor (TCR) and peptides presented by human leukocyte antigens (pHLA). The role of TCR–pHLA interactions in modulating T-cell metabolism is unknown. Here, for the first time, we explore the relative contributions of the main metabolic pathways to functional responses in human CD4^+^ and CD8^+^ T-cells. Increased expression of hexokinase II accompanied by higher basal glycolysis is demonstrated in CD4^+^ T-cells; cytokine production in CD8^+^ T-cells is more reliant on oxidative phosphorylation. Using antigen-specific CD4^+^ and CD8^+^ T-cell clones and altered peptide ligands, we demonstrate that binding affinity tunes the underlying metabolic shift. Overall, this study provides important new insight into how metabolic pathways are controlled during antigen-specific activation of human T-cells.

## Introduction

T-cells can be sub-divided into two main types by their expression of an accessory glycoprotein co-receptor, either CD4 or CD8, which facilitates their preferential interaction with MHC Class II or Class I molecules (HLA), respectively (1). CD4^+^ and CD8^+^ T-cells have divergent yet interacting roles related to immune homeostasis and pathogenesis of both communicable and non-communicable diseases. Effective functioning of CD4^+^ and CD8^+^ T-cells is energy demanding. The universal energy carrier adenosine 5′-triphosphate (ATP) in addition to assimilation and generation of biosynthetic precursors are required to initiate and sustain an immune response (2, 3). Immunometabolism describes how immune cells obtain ATP *via* differing rates of the energy-producing pathways and generate biosynthetic intermediates under quiescence and activation (4, 5). T-cell quiescence is associated with energy utilization *via* high-yield, slow burning metabolic processes dependent on fueling mitochondria for oxidative phosphorylation (6).

There is a burgeoning literature regarding T-cell metabolism, but with the exception of CD8^+^ T-cells (7–10), most data on T-cell metabolism are derived from mouse models and direct comparisons of human CD4^+^ and CD8^+^ T-cells have not been made. Murine CD4^+^ and CD8^+^ T-cells are bioenergetically similar when quiescent and are metabolically reprogrammed to a highly glycolytic metabolic state upon activation with CD8^+^ T-cells the more bioenergetic (11). Constitutive glycolytic metabolism results in long-lived effector T-cells in viral specific murine CD8^+^ T-cells (12). Activation is also accompanied by increased expression of GLUT1 and glycolysis pathway enzymes in both murine CD4^+^ and CD8^+^ T-cells (11, 13, 14). Surface levels of GLUT1 have been shown to identify human CD4^+^ and CD8^+^ T-cell with distinct characteristics. GLUT1^Hi^ T-cells produced elevated levels of IFNγ and had increased effector function (15). Naïve T-cell activation is linked to asymmetric division and the effector T-cell and memory T-cell that arise upon interaction with an antigen-presenting cell have metabolic differences. The effector T-cell is largely glycolytic, whereas the memory T-cell relies on oxidative metabolism governed by transcription factor c-myc (16). Post-infection, murine CD8^+^ memory T-cells retain a high spare respiratory capacity should re-infection occur (17). Increased glucose metabolism upon T-cell activation is critical for the rapid engagement of cellular proliferation, achieved *via* the generation of biosynthetic intermediate serine and downstream nucleotide production (2). Manipulating this pathway offers the potential to modulate regulatory T-cell differentiation and function (18, 19).

T-cell receptor (TCR) ligation to a peptide presenting HLA molecule (pHLA) is critical to the effective activation of T-cells (20, 21). The binding affinity between the TCR and core region of the peptide coupled with the half-life of peptide-TCR interaction collectively govern the downstream effector function (22, 23). The TCR-pHLA binding affinity confers underlying signaling cascades leading to an increased demand for the extracellular glucose needed to produce biosynthetic intermediates for proliferation in addition to cellular ATP (24, 25). Synthesis of metabolites, such as polyamines, cholesterol *via* fatty acids synthase, and pentose phosphate intermediates, has been shown to enhance T-cell activation (26, 27). To initiate and sustain this demand, hematopoietic cells generally exhibit a “Warburg-like” switch to glycolysis (28). The reliance of human CD8^+^ T-cells on glycolysis when stimulated with natural ligands (Epstein–Barr Viral peptides) has been reported (7); how TCR-pHLA binding affinity might control the corresponding metabolic response in human T-cells is unknown. Murine CD8^+^ T-cells show TCR binding affinity-dependent induction of IRF4 and downstream metabolic control (29).

This is the first study to investigate the metabolic tuning that occurs in human T-cells upon activation *via* the TCR and includes consideration of the role of TCR-pHLA binding affinities. Stimulation with native peptide provides a more physiologically relevant mechanism of T-cell activation compared to anti-CD3/anti-CD28. Furthermore, cytokine production by both CD4^+^ and CD8^+^ T-cells is shown to depend on glycolysis with differential mitochondrial dependence between these T-cell subsets.

## Materials and Methods

### Human CD4^+^ and CD8^+^ T-Cell Isolation

Human peripheral blood was collected between 0830 hours and 1000 hours from healthy, non-fasted individuals into heparinised Vacuettes™ (Greiner Bio-one, Frickenhausen, Germany) and processed within 10 min of collection. All samples were collected with informed written consent and ethical approval was obtained from Wales Research Ethics Committee 6 (13/WA/0190).

Mononuclear cells (MNCs) were isolated by layering whole blood (1:1) onto Histopaque (Sigma-Aldrich, Poole, UK) prior to centrifugation at 805*g* for 20 min at room temperature. MNCs were removed and washed with RPMI 1640 (Life Technologies, Paisley, UK) twice by centrifugation at 515*g*. The MNC pellet was resuspended in media specific for the downstream assay and cell density determined using the Countess^®^ automated cell counter (Life Technologies).

CD4^+^ or CD8^+^ T-cells were isolated *via* a negative selection process using magnetic microbeads as described by the manufacturer (autoMACS; Miltenyi Biotec, Cologne, Germany). Purity of individual populations was monitored using flow cytometry and was typically >90%. For non-matched T-cell experiments, the mean ± SD donor age for CD4^+^ T-cell preparations was 39.2 ± 14.68 years (*n* = 12; 7 females and 5 males) and for CD8^+^ T-cells was 35.1 ± 13.21 years (*n* = 16; 7 females and 9 males).

### T-Cells

T-cell clones, DCD10, and ILA1 were created (30, 31) and passaged as previously described (32). Briefly, clones were expanded with irradiated (3,100 Gy) PBMCs from three donors in R10 [RPMI 1640 supplemented with 10% FBS, 100 U/ml penicillin, 100 µg/ml streptomycin, 1× MEM non-essential amino acid, 1 mM sodium pyruvate, 10 mM HEPES buffer (Life Technology)] with 20 IU/ml of IL-2 (Aldesluekin, Proleukin, Prometheus, San Diego, CA, USA) and 1 µg/ml of phytohaemaglutinin (Alere, Cheshire, UK). Additionally, ILA1 was cultured with 25 ng/ml of IL-15 (PeproTech, Rocky Hill, NJ, USA) and IL-2 increased to 200 IU/ml 7 days post expansion. For this purpose of this study, clones were used spanning 3–4 passages. Prior to performing assays, clones were washed from culture media and rested in R5 (as for R10 with 5% FBS) for 24 h. Peptides (Peptide Protein Research Limited, Fareham, UK) were synthesized to greater than 95% purity, stored as 20 mM stocks at −80°C in DMSO and working aliquots made to 1 mM with R0 (as for R10 but with no FBS) and stored at −20°C or 4°C.

### Metabolic Analysis

Metabolic analysis was carried out using an Extracellular Flux Analyzer XF^e^24 (Seahorse Bioscience). Briefly, 0.25 × 10^6^ cells were seeded onto a Cell-Tak (Corning)-coated microplate allowing the adhesion of T-cells. Mitochondrial stress and glycolytic parameters were measured *via* oxygen consumption rate (OCR) (pmoles/min) and extracellular acidification rate (ECAR) (mpH/min), respectively, with use of real-time injections. For mitochondrial stress, cells were resuspended in XF assay media supplemented with 5.5 mM glucose and 1 mM pyruvate and injections oligomycin (0.75 µM), carbonyl cyanide-*4*-(trifluoromethoxy)phenylhydrazone (FCCP; 1 µM) and rotenone and antimycin (both 1 µM) were used. For glycolysis, cells were resuspended in XF assay media with use of injections glucose (11.1 mM), oligomycin (0.75 µM) and 2-deoxy-d-glucose (100 mM). Respiratory parameters were calculated as previously described (33). All chemicals were purchased from Sigma unless stated otherwise. Calculations for individual metabolic parameters can be found as described previously (33) or per manufacturer’s instructions (Seahorse Bioscience).

#### Activation

To monitor the glycolytic switch upon activation, CD4^+^ and CD8^+^ T-cells were resuspended in serum-free XF Assay media supplemented with 11.1 mM glucose and 2 mM l-glutamine (Sigma). ECAR and OCR were measured simultaneously throughout the experiment, i.e., 1 h before activation and 4 h after. T-cells were activated *via* the multi-injection port with anti-CD3 (0.2 µg/ml; HIT3a, BioLegend) and CD28 (20 µg/ml; CD28.2, BioLegend). A final injection of 2-DG (100 mM) was used to immediately arrest glycolysis. Isotype controls, mIgG2a κ (0.2 µg/ml; MOPC-173, BioLegend) and mIgG1 κ (20 µg/ml; MOPC-21; BioLegend) were used. The OCR/ECAR ratio was calculated by dividing the eight corresponding OCR and ECAR measurements pre- (dotted boxes) or post- (dashed boxes) antibody injection. Fold ECAR change was calculated by dividing the single point post antibody injection by the single point pre antibody injection. Peptide stimulation relied on the cross presentation of specific peptides by corresponding T-cell clones.

#### Inhibition

Baseline ECAR of CD4^+^ and CD8^+^ T-cells was determined for roughly 1 h prior to injection of GLUT1/4 inhibitor ritonavir (20 µM; Sigma). A 40-min period of incubation with the inhibitor occurred prior to injection of αCD3/28 as above. Corresponding ECAR was monitored for 4 h after αCD3/28 injection. A final injection of 2-DG (100 mM) arrested glycolysis. Fold ECAR change was calculated by dividing the 13 measurements post antibody injection (dashed box) by the 13 measurements pre antibody injection (dotted box).

### Flow Cytometry

Purity of CD4^+^, or CD8^+^ T-cells was monitored using flow cytometry. Briefly, 2.5 × 10^5^ cells were left unstained or incubated with anti-CD4^+^ AlexaFluor^®^647 (mIgG2b, clone OKT4, eBioscience) or anti-CD8^+^ PE (mIgG1, clone HIT8a, eBioscience) using standard techniques. Cells were acquired (FACSAria I, BD Biosciences) and downstream analysis was with FlowJo version 1.3 (Tree Star, OR, USA). To assess mitochondrial content, MNCs were stained with the mitochondrial probe MitoTracker Green (Life Technologies). MNCs (5 × 10^5^ cells) were incubated with 20 nM MitoTracker Green for 30 min at 37°C then surface labeled with lineage markers as above before acquisition and analysis. T-cell activation was monitored by expression of CD69 (mIgG1, FN50, BioLegend), flow cytometry plots are representative of live cells with dead cell exclusion performed *via* DRAQ7 (1 µM; Biostatus, UK).

### Effect of Respiratory Inhibitors on Cytokine Output

CD4^+^ and CD8^+^ T-cells were cultured at 0.5 × 10^6^ cells/500 μl of phenol red free RPMI (Sigma) + 2 mM GlutaMAX (ThermoFisher). T-cells were cultured with 2-deoxy-d-glucose (25 mM) or oligomycin (1 µM) at 37°C in 5% CO_2_-in-air for 24 h. All chemicals were purchased from Sigma. To prevent impaired T-cell activation, after 3 h 5% fetal bovine serum (FBS, HyClone, ThermoFisher Scientific) was added. Cells were analyzed *via* flow cytometry for cell death (DRAQ7) and activation (CD69); the supernatant was removed and stored at −20°C for downstream cytokine analysis. IFNγ and IL-2 were analyzed using ELISA as per manufacturer’s instructions (DuoSets; R&D Systems).

### Immunoblot

CD4^+^ and CD8^+^ T-cell lysate proteins were quantified using the DC Assay (Bio-Rad, Hemel Hempstead, UK) and separated (10 mg per lane) using 10% (vol/vol) SDS-polyacrylamide gel electrophoresis, with molecular weight markers in parallel lanes (Bio-Rad). After electrophoresis, proteins were transferred to a polyvinylidene difluoride membrane (Bio-Rad); non-specific binding was blocked using 5% (wt/vol) bovine serum albumin (BSA; Sigma) in Tris-buffered saline (Sigma) for 1 h at room temperature. Membranes were probed with rabbit monoclonal antibodies targeting glucose transporter 1 (GLUT1; ab115730; Abcam), hexokinase I (HKI; 2024), hexokinase II (HKII; 2867), phosphofructokinase (PFKP; 8164), glyceraldehyde-3-phosphate dehydrogenase (GAPDH; 5174), pyruvate kinase (PKM2; 4053) lactate dehydrogenase (LDH; 3582), total S6 ribosomal protein (2217), and phospho-S6 ribosomal protein (Ser235-236; 4858). Protein loading was evaluated and normalized using mouse monoclonal antibody targeting β-actin expression (3700). All antibodies were purchased from Cell Signaling unless otherwise stated (Danvers, MA, USA). Primary antibodies were used at 1:1,000 dilutions in Tris-buffered saline, 0.1% Tween 20 (pH 7.6; Sigma) overnight at 4°C. Membranes were washed and incubated in either anti-rabbit or anti-mouse horseradish peroxidase-conjugated secondary antibody (Cell Signaling) in 5% (wt/vol) BSA in Tris-buffered saline for 1.5 h, and then washed. Steady-state levels of immunoreactive proteins were visualized using enhanced chemiluminescence (Western C, Bio-Rad), and densitometry on non-saturated immunoblots was measured using ImageJ software (FIJI). Full immunoblots are shown in supplementary material.

### Data Analysis

Statistical analysis was performed using GraphPad Prism version 6 (USA). Data are represented as the mean + SEM. A non-paired *t*-test was used for the different metabolic data, densitometry immunoblots, and metabolic inhibition comparisons. One-way ANOVA was used to compare 24 h activated T-cells samples and altered peptide ligand (APL) ECAR and OCR fold change. Statistical analysis was performed on the technical repeats when considering the clone data. Significant values were taken as *p ≤ 0.05, **p ≤ 0.01, ***p ≤ 0.001.

## Results

### CD4^+^ T-Cells Have a Greater Glycolytic Potential than CD8^+^ T-Cells

**Figure 1:**
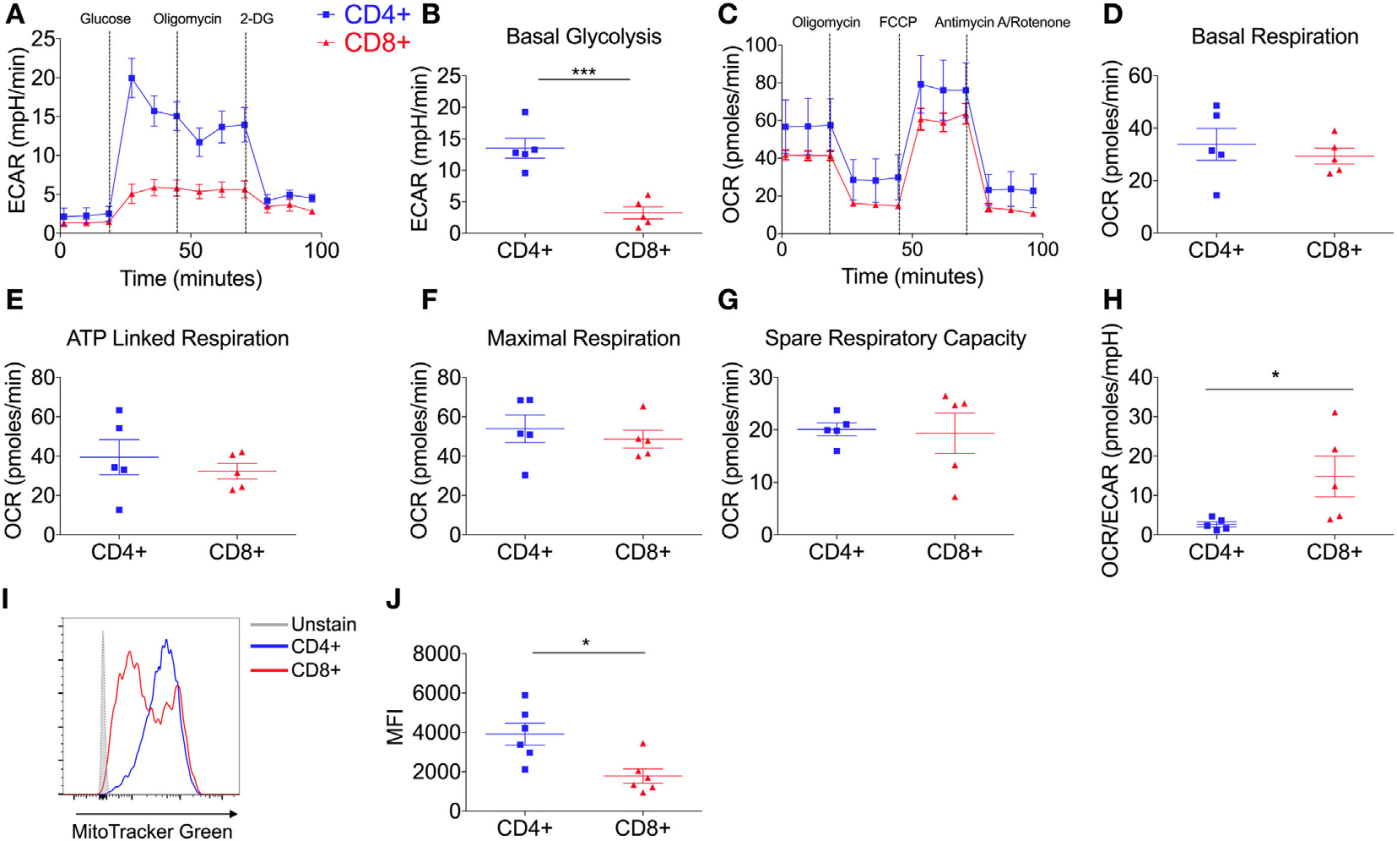
CD4^+^ T-cells are more glycolytic than CD8^+^ T-cells. **(A)** Glycolysis by CD4^+^ and CD8^+^ T-cells isolated from non-matched donors was measured using extracellular acidification rate (ECAR) with injections glucose (11.1 mM), oligomycin (0.75 µM), and 2-DG (100 mM); including parameter **(B)** basal glycolysis calculated by subtracting the three averaged measurements after glucose injection from the non-glycolytic acidification. **(C)** Oxidative phosphorylation profiles of CD4^+^ and CD8^+^ T-cells measured by the oxygen consumption rate (OCR) with injections oligomycin (0.75 µM), FCCP (1 µM) and antimycin A/rotenone (both 1 µM); including OXPHOS parameters **(D)** basal respiration, **(E)** ATP-linked respiration, **(F)** maximal respiration and **(G)** spare respiratory capacity. **(H)** OCR/ECAR ratio of CD4^+^ and CD8^+^ T-cells (pmoles/mpH) as determined *via* the division of basal respiration and basal glycolysis parameters. Mitochondrial content of CD4^+^ and CD8^+^ T-cells was assessed by flow cytometry with MitoTracker Green and **(I)** a representative example is shown and **(J)** summary data for *n* = 6. Data are from five **(A–H)** non-matched donors, and six **(J)** matched independent experiments. Data expressed as mean ± SEM; *p ≤ 0.05, ***p ≤ 0.001.

To investigate the glycolytic potential of human T-cells, we undertook bioenergetics analysis of total, non-matched CD4^+^ versus CD8^+^ T cells. ECAR was measured and showed that all glucose starved T-cells responded to glucose injection with increased ECAR but failed to show a further increase after injection of the ATP synthase inhibitor, oligomycin (Figure 1A). There was no significant difference in the non-glycolytic acidification between CD4^+^ and CD8^+^ T-cells (Figure S1A in Supplementary Material). Most notably, CD4^+^ T-cells exhibited significantly higher levels of basal glycolysis compared to CD8^+^ T-cells (Figure 1B). Oxidative phosphorylation profiles of CD4^+^ and CD8^+^ T-cells were also determined using extracellular flux analysis for oxygen consumption rate (OCR; Figure 1C). CD4^+^ and CD8^+^ T-cells did not differ in rates of basal, maximal or ATP-linked respiration, spare respiratory capacity (Figures 1D–G) or non-mitochondrial respiration and proton leak (Figures S1B,C in Supplementary Material). The combined changes in OCR (basal respiration) and ECAR (basal glycolysis) give CD4^+^ T-cells a significantly lower OCR/ECAR ratio than CD8^+^ T-cells (Figure 1H). Analysis of mitochondrial content using flow cytometry with the mitochondrial stain MitoTracker Green, revealed that donor-matched CD4^+^ T-cells have a significantly higher mitochondrial content than CD8^+^ T-cells (Figures 1I,J) in agreement with findings in murine models (11).

### CD4^+^ T-Cells Express High Levels of Hexokinase Isoforms

**Figure 2:**
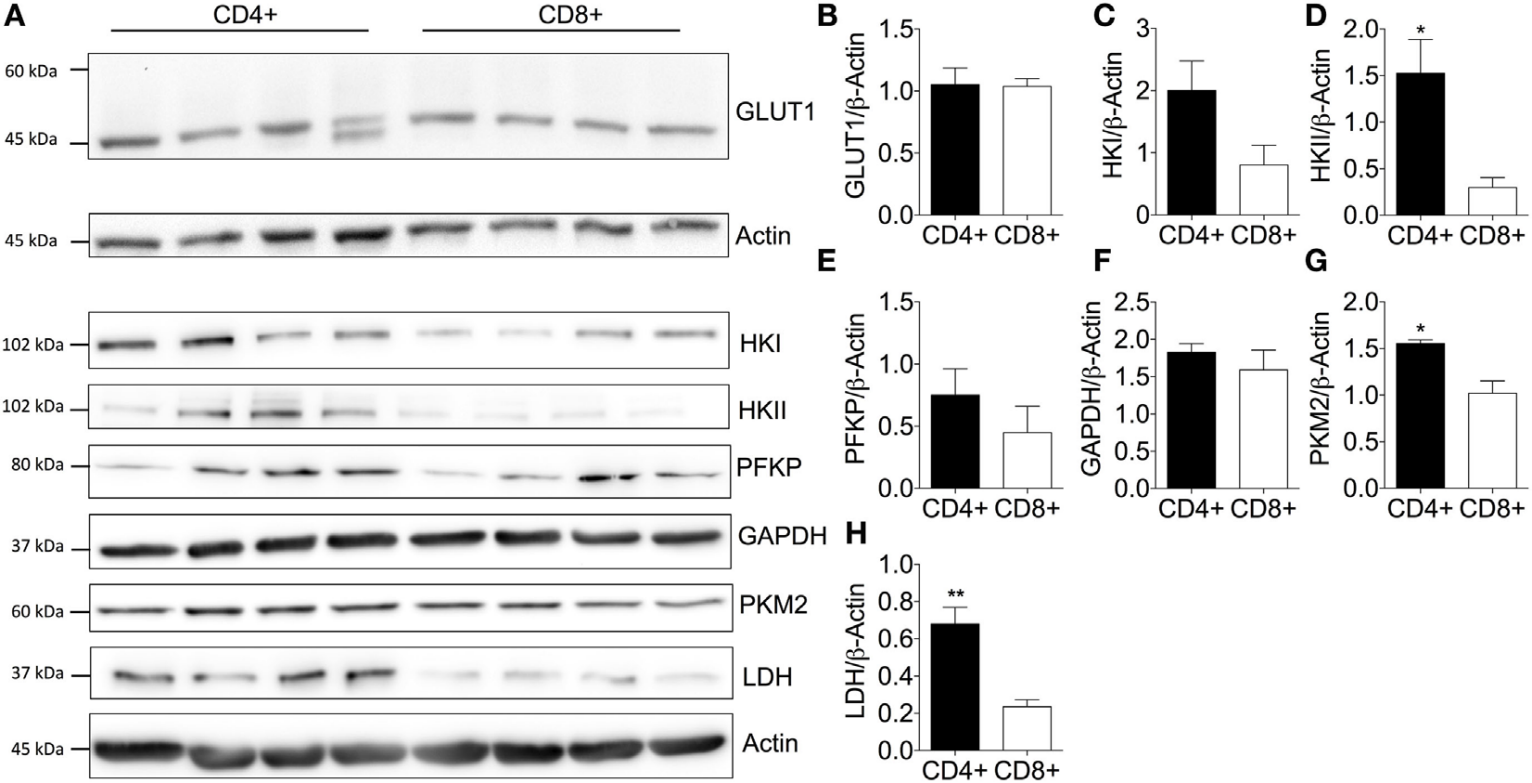
Elevated expression of key glycolytic enzymes enhances basal glycolysis of CD4^+^ T-cells. **(A)** Protein immunoblot and respective densitometry showing **(B)** GLUT1, **(C)** hexokinase I (HK), **(D)** hexokinase II, **(E)** phosphofructokinase (PFKP), **(F)** glyceraldehyde-3-phosphate dehydrogenase (GAPDH), **(G)** pyruvate kinase (PKM2), and **(H)** lactate dehydrogenase (LDH) expression levels between basal CD4^+^ and CD8^+^ T-cells. Full scan blots are shown in Figure S9 in Supplementary Material. Data are from four independent experiments. Data expressed as mean + SEM; *p ≤ 0.05, **p ≤ 0.01.

To investigate the underlying cause for increased glycolytic metabolism by quiescent CD4^+^ T-cells, key transporters and enzymes within the glycolysis pathway were analyzed (Figure 2A): GLUT1 as the predominant glucose transporter in human and murine CD4^+^ T-cells (13); hexokinase (HK) I and II that catalyze the transfer of phosphate from ATP to glucose thereby consuming one molecule of ATP and ‘trapping” glucose in the cell (34); phosphofructokinase (PFKP) which catalyzes a rate-limiting reaction that consumes a second ATP molecule; GAPDH which is critical to the production of two ATP molecules and two nicotinamide adenine dinucleotide molecules (NADH + H^+^) (35); pyruvate kinase (PKM2) that catalyzes the final rate-limiting step of glycolysis to produce two ATP molecules per glucose; and lactate dehydrogenase (LDH) that converts pyruvate into lactate yielding the protons measured as ECAR.

Immunoblotting of CD4^+^ and CD8^+^ T-cells from non-matched donors revealed that GLUT1 levels were consistent between CD4^+^ and CD8^+^ T-cells (Figure 2B). A double band observed in one of the CD4^+^ T-cell donors could be due to altered glycosylation status of the GLUT1 protein as observed in other human glucose transporters (36, 37). HKII (Figure 2D), PKM2 (Figure 2G), and LDH (Figure 2H) were all increased in CD4^+^ T-cells and likely underpin the increased glycolytic capacity of this population compared to CD8^+^ T-cells. There were no differences in HKI (Figure 2C), PFKP (Figure 2E), or GAPDH (Figure 2F). However, we cannot rule out differential kinetics of any of these glycolytic enzymes between the two T-cell populations and this should be considered in future experiments.

### CD4^+^ and CD8^+^ T-Cells Increase Glycolytic Flux upon Stimulation

**Figure 3:**
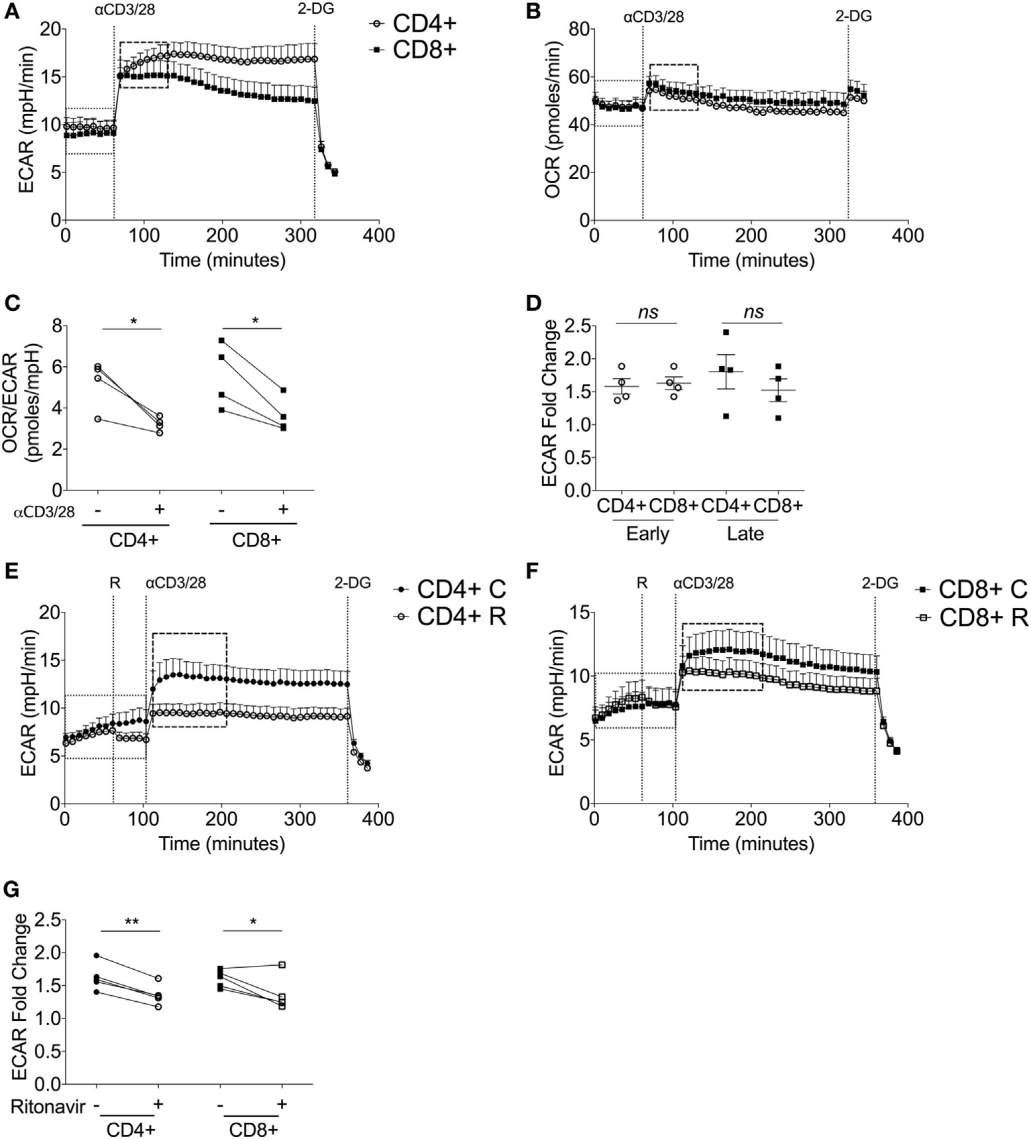
Upon stimulation, both CD4^+^ and CD8^+^ T-cells switch to glycolytic metabolism. **(A)** Extracellular acidification rate (ECAR) and **(B)** oxygen consumption rate (OCR) of donor-matched CD4^+^ and CD8^+^ T-cells upon stimulation with antibodies, anti-CD3 (0.2 µg/ml), and anti-CD28 (20 µg/ml). 2-deoxy-d-glucose (100 mM) was added at the end of the experiment to immediately arrest glycolysis. **(C)** OCR/ECAR ratio pre- and post- activation calculated by dividing OCR by ECAR (dotted box; pre, dashed box; post). **(D)** ECAR fold change of early and late glycolytic changes comparing CD4^+^ and CD8^+^ T-cells. ECAR of **(E)** CD4^+^ T-cells and **(F)** CD8^+^ T-cells ± ritonavir (20 µM) prior to injection of activating antibodies as above with a final injection of 2-DG (100 mM). *C, control; R, ritonavir*. **(G)** Fold change calculated *via* division of the dotted boxes of CD4^+^ and CD8^+^ T-cells ± ritonavir. Data are from four donor-matched **(A–D)** or five donor-matched independent experiments **(E–G)**. Graphed points represent averaged data from the four/five independent experiments. Data expressed as mean + SEM; *p ≤ 0.05, **p ≤ 0.01, *ns*, not significant.

Having established that human CD4^+^ T-cells are more glycolytic than CD8^+^ T-cells during quiescence, we then investigated the metabolic plasticity of both subsets in response to stimulation. Donor-matched CD4^+^ and CD8^+^ T-cells were activated with anti-human CD3 and anti-human CD28 antibodies and both ECAR and OCR monitored for a period of 30 cycles using extracellular flux analysis (4.13 h; Figures 3A,B).

Immediately upon injection of anti-CD3/CD28, both CD4^+^ and CD8^+^ T-cells increase ECAR indicating heightened glycolysis (Figure 3A). This was accompanied by a slight increase in OCR (Figure 3B). Isotype controls had no effect on either glycolysis or oxygen consumption (Figures S2A,B in Supplementary Material). Elevated ECAR levels were maintained by CD4^+^ and CD8^+^ T-cells for the duration of the experiment. Calculation of the OCR/ECAR ratio revealed a significant immediate increase in glycolytic flux in both CD4^+^ and CD8^+^ T-cells upon activation (Figure 3C). The early engagement of glycolysis, represented as a fold ECAR change, was homologous in CD4^+^ and CD8^+^ T-cells (Figure 3D). There was a temporal decline in glycolysis by CD8^+^ T-cells, whereas this response was sustained in CD4^+^ T-cells (Figure 3D). To determine the role of glucose transporters in the activation-dependent glycolytic switch, ritonavir, an inhibitor of both GLUT1 and GLUT4 (38), was used. Ritonavir significantly dampened activation-induced glycolysis in both CD4^+^ and CD8^+^ T-cells (Figures 3E–G).

The potential contribution of key enzymes to an activation-dependent metabolic switch was then determined by comparing expression at 24 h with or without exposure to anti-CD3/CD28 (Figures S3A–G in Supplementary Material). Activation did not affect levels of HKI in donor-matched CD4^+^ or CD8^+^ T-cells (Figure S3B in Supplementary Material), whereas HKII was markedly increased in both CD4^+^ and CD8^+^ T-cells (Figure S3C in Supplementary Material). PFKP and GAPDH expression also increased upon activation of both cell types (Figures S3D,E in Supplementary Material). PKM2 levels were increased in CD4^+^ T-cells upon activation but remained constant in CD8^+^ T-cells (Figure S3F in Supplementary Material). Levels of LDH were increased in both subsets upon activation with the response by CD8^+^ T-cells significant (Figure S3G in Supplementary Material). These experiments indicate that the immediate metabolic switch upon activation is probably dependent on glucose transport *via* GLUT1. Subtle differences in the expression and probable activity of GLUT1 and different glycolytic enzymes contribute to altered kinetics of response by CD4^+^ versus CD8^+^ T-cells.

### Increased GLUT1 Expression Facilitates CD4^+^ and CD8^+^ T-Cell Activation

**Figure 4:**
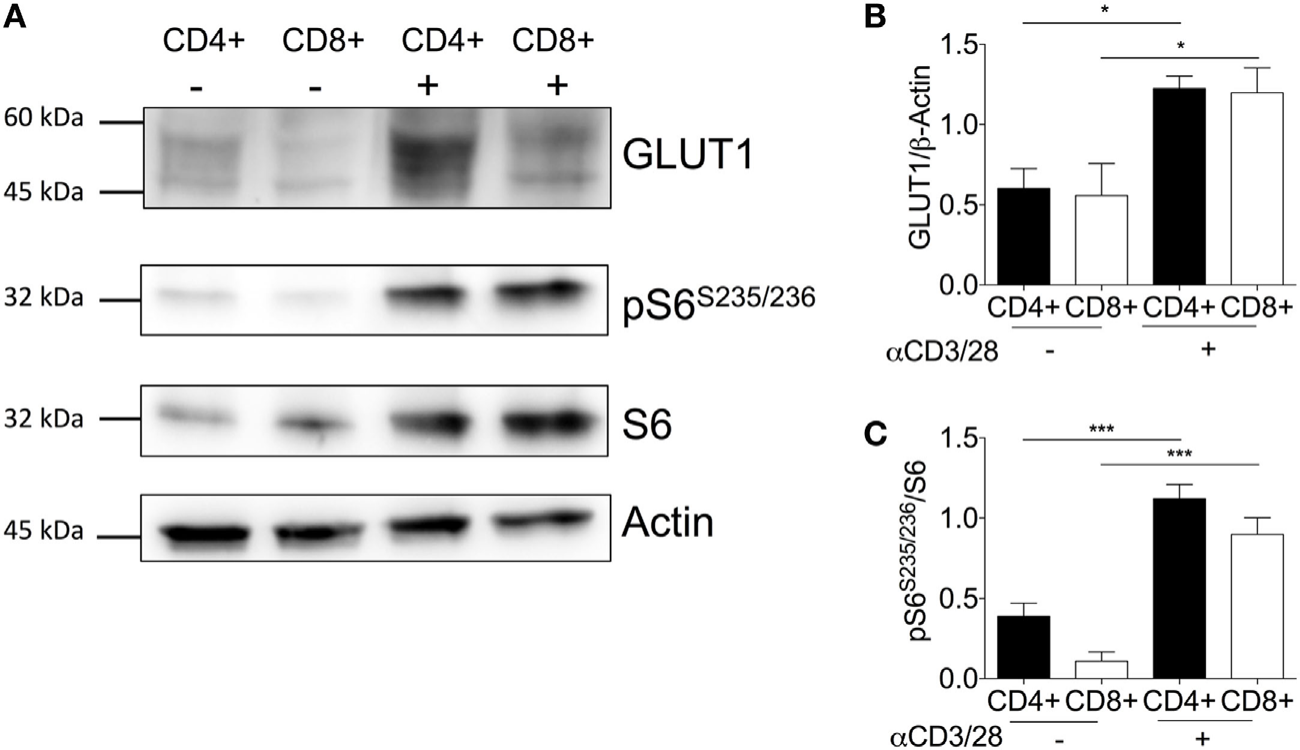
Activation is associated with increased GLUT1 expression in CD4^+^ and CD8^+^ T-cells. **(A)** Protein immunoblot representative of one matched donor, and respective densitometry of all donors showing, **(B)** GLUT1, **(C)** ribosomal proteins; phospho-S6 and total S6 expression levels between unstimulated(−) and activated(+) (anti-CD3; 2 µg/ml and anti-CD28; 20 µg/ml) CD4^+^ and CD8^+^ T-cells for 24 h. Full scan blots are shown in Figure S10 in Supplementary Material. Data are from five independent experiments with donor-matched CD4^+^ and CD8^+^ T-cells **(A–C)**. Data expressed as mean + SEM; *p ≤ 0.05, ***p ≤ 0.001.

To further explore the contribution of glucose transporters to enhanced glycolysis upon activation, GLUT1 expression before and after activation was analyzed by immunoblotting (Figure 4A). GLUT1 expression was significantly increased upon activation in donor-matched CD4^+^ and CD8^+^ T-cells (Figure 4B). The *de novo* synthesis of proteins such as cytokines requiring transcription and translation is a key feature of T-cell effector responses. Therefore, we next assessed the levels of downstream mTORC1 target, ribosomal S6, a protein involved in the translation of 5′TOP mRNAs (39, 40) (Figure 4A). There was an increase in phosphorylated ribosomal protein S6 (pS6Ser235/236), with ratio of pS6/S6 significantly different between unactivated and activated T-cell subsets (Figure 4C). A marked increase in translation *via* increased phosphorylated S6 would support the production of cytokines to mount a successful immune response. This is feasible as a marked decrease in pS6 and mTORC1 activity upon triple therapy strategies reduces murine T-cell effector function thus decreasing allograft rejection (41, 42).

### CD8^+^ T-Cells Are Dependent on Mitochondrial Metabolism for Cytokine Production

**Figure 5:**
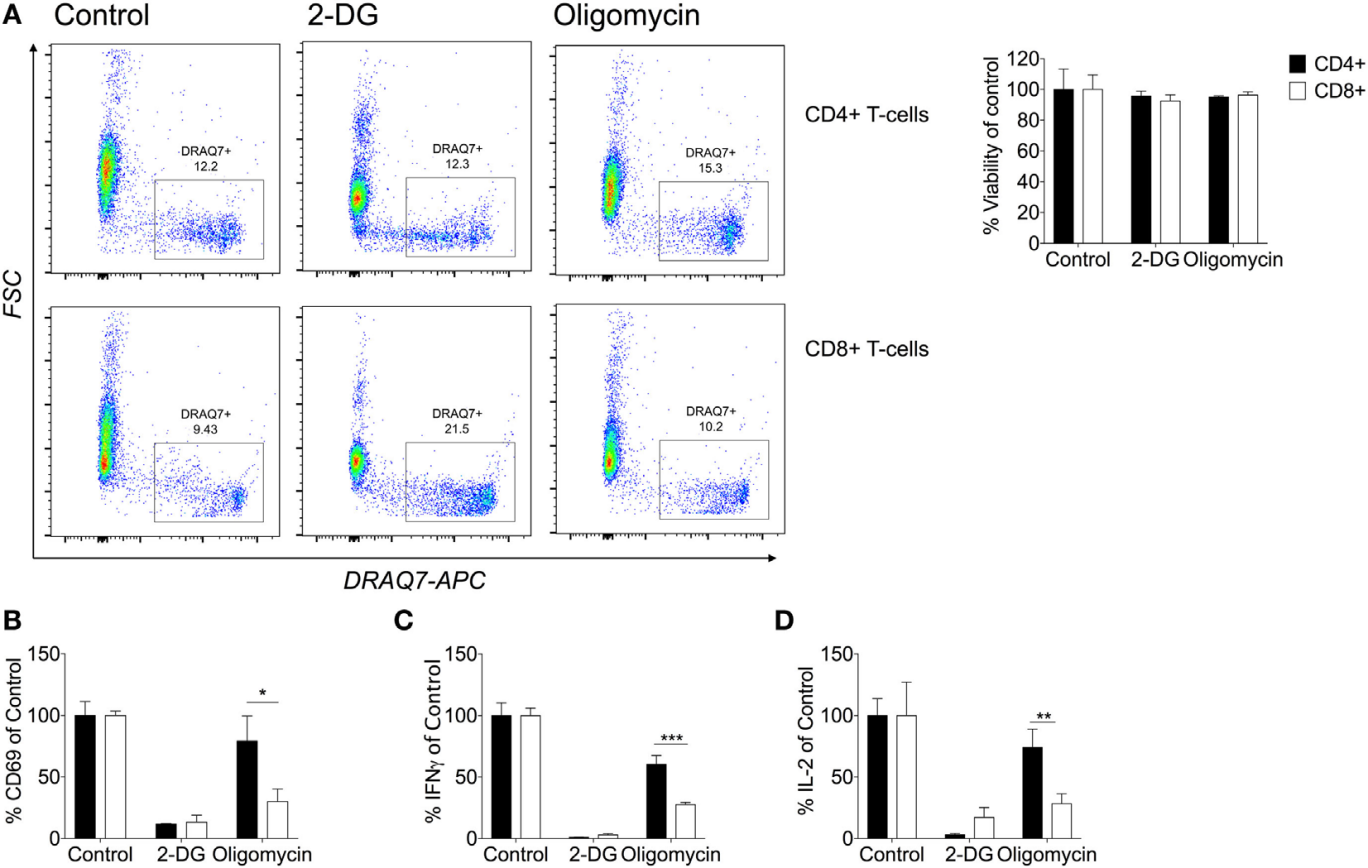
CD8^+^ T-cells are dependent on their mitochondria for cytokine production. **(A)** Viability was determined by DRAQ7 (1 µM) flow cytometry of CD4^+^ and CD8^+^ T-cells stimulated with anti-CD3 (2 µg/ml) and anti-CD28 (20 µg/ml) for 24 h in the presence of 2-deoxy-d-glucose (2-DG; 25 mM), or oligomycin (1 µM). Viability expressed as a percentage of the 24 h activated control **(B)** Percentage CD69 of CD4^+^ and CD8^+^ T-cells (DRAQ7-negative) relative to control. The effects of metabolic inhibitors 2-DG and oligomycin were considered on percentage cytokine production of **(C)** IFNγ and **(D)** IL-2 comparison of CD4^+^ and CD8^+^ T-cells. Data are representative of three to five independent experiments. Data expressed as mean + SEM; *p ≤ 0.05, **p ≤ 0.01, ***p ≤ 0.001.

Currently, little is known about the metabolic pathways utilized by human CD4^+^ and CD8^+^ T-cells and whether they differ upon effector function. In mice, NK cells require both glycolysis and oxidative phosphorylation for IFNγ production (43). Understanding the metabolic pathways that contribute to downstream cytokine production might offer potential therapeutic targets. Therefore, the role of glycolysis and oxidative phosphorylation were considered by activating cells with anti-CD3/CD28 in the presence of the metabolic inhibitors: 2-DG which inhibits glycolysis, and oligomycin which inhibits oxidative phosphorylation. Cell death was monitored using DRAQ7 by flow cytometry with neither inhibitor having an effect on non-matched CD4^+^ or CD8^+^ T-cell viability (Figure 5A). Activation was monitored through CD69 expression for both subsets, whereby inhibition of glycolysis but not oxidative phosphorylation was associated with reduced expression of CD69 indicating decreased activation of both CD4^+^ and CD8^+^ T-cells if glycolysis is abrogated (Figure 5B). Inhibition of oxidative phosphorylation, however, only reduced CD69 expression in CD8^+^ T-cells, indicating a greater contribution of mitochondrial-dependent mechanisms to activation of these cells (Figure 5B).

In agreement with other studies, we have shown that limiting glycolysis with 2-DG inhibited production of IFNγ and IL-2 in CD4^+^ and CD8^+^ T-cells (Figures 5C,D) (7). IFNγ and IL-2 were not detectable in the unstimulated samples (data not shown). The contribution of oxidative phosphorylation to the expression of CD69 and the production of both IFNγ and IL-2 production was significantly greater in CD8^+^ than CD4^+^ T-cells. To our knowledge, this is the first time that different metabolic requirements of human CD4^+^ and CD8^+^ T-cells have been demonstrated.

We also found that this difference in metabolic requirement was evident for wider cytokine production. The production of IL-13, IL-17, and IL-10 by CD4^+^ T-cells (Figures S4A–D in Supplementary Material) was reduced significantly after incubation with 2-DG; the decrease in IL-4 was not significant. Only the Th2 cytokines, IL-4 and IL-13, and to a lesser extent IL-10, were decreased by inhibition of oxidative phosphorylation. For CD8^+^ T-cells, granzyme B and MIP-1β, like IL-2 and IFNγ, were reduced upon treatment with either 2-DG or oligomycin, confirming a greater contribution of mitochondrial metabolism to CD8^+^ T-cell cytokine and granzyme B production (Figures S5A,B in Supplementary Material). Similar to our findings, murine CD8^+^ T-cells are more resistant than CD4^+^ T-cells to knockout of GLUT1 (11). These data further support differential metabolic kinetics of both T-cell subsets that could be important in nutrient competitive and restricted environments.

### TCR Peptide HLA Induced T-Cell Activation Stimulates Greater Glycolytic Flux Compared to Non-Natural CD3/CD28 Stimulation

**Figure 6:**
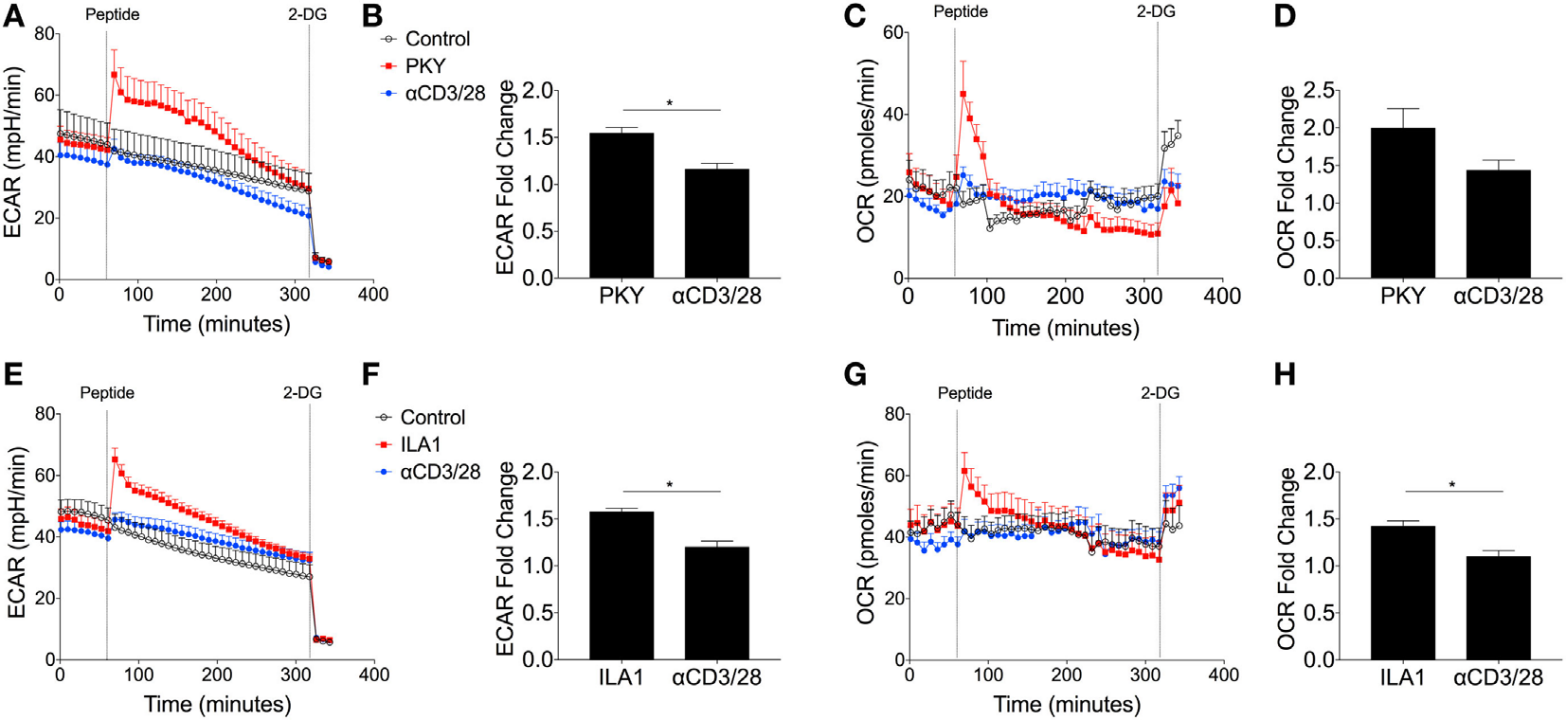
Metabolic response of CD4^+^ DCD10 and CD8^+^ ILA T-cell clones to native peptides. Metabolic analysis of CD4^+^ T-cell clone, DCD10 in response to native Flu1 HA_306–318_, peptide, with sequence; PKYVKQNTLKLAT (PKY). **(A)** Comparative extracellular acidification rate (ECAR) stimulation of DCD10 CD4^+^ T-cell clone with PKY (10 µM) and anti-CD3 (0.2 µg/ml) and anti-CD28 (20 µg/ml). Final injection of 2-deoxy-d-glucose (100 mM). **(B)** Fold ECAR change calculated with use of single measurement prior to peptide injection and single measurement after peptide injection. **(C)** Corresponding oxygen consumption rate (OCR) and **(D)** OCR fold change after stimulation with PKY or anti-CD3 anti-CD28. **(E)** Metabolic analysis *via* ECAR of CD8^+^ T-cell clone, ILA1 with ubiquitous tumor-associated antigen human telomerase reverse transcriptase (hTERT_540–548_) peptide ILA1 (ILAKFLHWL; 10 µM) with final injection of 2-DG (100 mM). **(F)** ECAR fold change as calculated previously. **(G)** Corresponding OCR and **(H)** OCR fold change after stimulation with ILA1 or anti-CD3 anti-CD28. Data are representative of four to twelve technical repeats comprising of two to three independent experiments. Data expressed as mean + SEM; *p ≤ 0.05.

Most studies of T-cell metabolism use anti-CD3/CD28 stimulation to activate T-cells rather than natural ligands although there are a few notable exceptions (7, 29). To address these shortcomings here, for the first time, antigen-specific human T-cell clones were used to investigate T-cell metabolism after stimulation directly through the antigen-specific TCR-pHLA interaction. Two T-cell clones were used: DCD10, a HLA-DRB*0101 restricted, influenza hemagglutinin (HA_306–318_: PKYVKQNTLKLAT) specific CD4^+^ T-cell clone; and ILA1, a HLA-A*0201-restricted, tumor-associated antigen human telomerase reverse transcriptase (hTERT_540–548_: ILAKFLHWL) specific CD8^+^ T-cell clone. Extracellular flux analysis relied on the presentation of peptide within T-cell populations, whereby the peptide initially binds to the HLA complex. Initial experiments were performed to optimize peptide concentrations for both clones (Figures S6A–D in Supplementary Material). We confirmed the expression of HLA-DR and MHC class I of DCD10 by flow cytometry (Figure S7A in Supplementary Material). In addition the ability of DCD10 to cross-present peptide was monitored *via* ELISA, whereby overnight cultures with native peptide PKY induced production of IFNγ and MIP-1β in comparison to negative peptide (5T4; Figure S7B in Supplementary Material). The response of the CD4^+^ DCD10 clone to the PKYVKQNTLKLAT peptide (PKY) was monitored by extracellular flux analysis in comparison with activating antibodies, anti-CD3, and anti-CD28. Both PKY and anti-CD3/anti-CD28 induced an immediate increase in ECAR (albeit to a lesser extent in anti-CD3/anti-CD28 stimulated cells), with 2-DG injection confirming the role of glycolysis (Figure 6A). Stimulation of DCD10 to its natural ligand PKY induced a significantly greater ECAR fold change in comparison to anti-CD3/anti-CD28 (Figure 6B). The corresponding OCR revealed a substantial increase in oxygen consumption upon injection of PKY, less so with anti-CD3/anti-CD28 (Figure 6C). PKY induced a twofold increase in oxygen consumption upon peptide stimulation, whereas anti-CD3/anti-CD28 induced roughly a 1.5-fold change in oxygen consumption (Figure 6D). Dual increases in both glycolysis and oxidative phosphorylation (represented as increased ECAR and OCR) *via* use of PKY proved that the use of antigen specificity is a viable option for extracellular flux analysis. We also altered the DCD10 native peptide (PKY) at residue number 11 from threonine to arginine and determined the metabolic response (Figure S8 in Supplementary Material). Here, we found that the residue alteration at position 11 had no effect on peptide recognition for the DCD10 clone using extracellular flux analysis, with ECAR and OCR remaining unchanged (Figure S8A–E in Supplementary Material).

We also compared the ILAKFLHWL index peptide and its metabolic effect on T-cell clone, ILA1. Following index peptide injection, ILA1 also exhibited significantly enhanced glycolysis (Figure 6E), in comparison to anti-CD3/anti-CD28 stimulation. This observation was reflected in the ECAR fold change (Figure 6F). The oxygen consumption was also monitored throughout the experiment (Figure 6G), which revealed a significant 1.5-fold increase after ILA1 peptide interaction, whereas there was no notable OCR increase in anti-CD3/anti-CD28-treated ILA1 clones (Figure 6H). Collectively, these data show that naturally recognized peptides produce a different metabolic signature compared to anti-CD3/anti-CD28 stimulation.

### TCR Binding Affinity Governs Differential Glycolytic Thresholds in Antigen-Specific T-Cells

**Figure 7:**
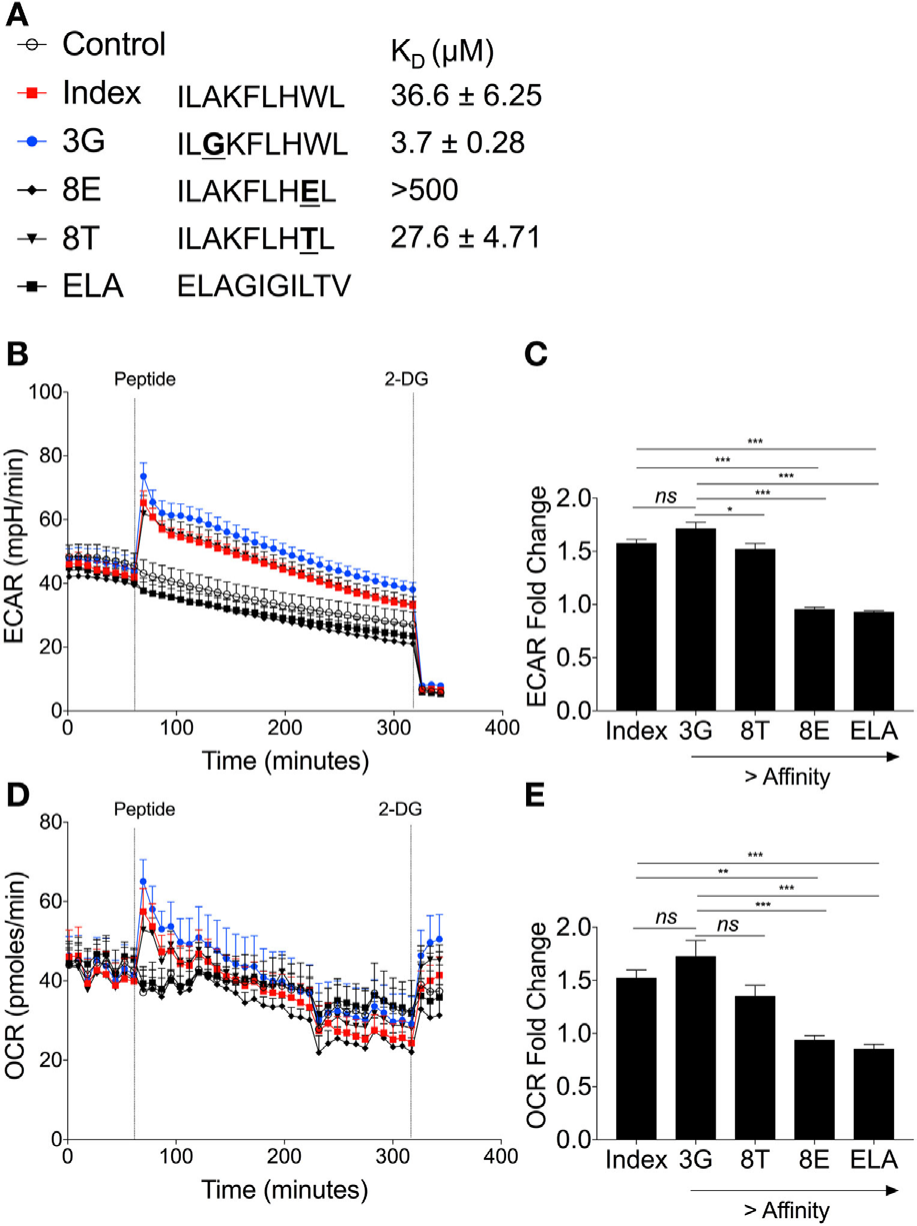
Peptide binding affinity affects underlying metabolic changes in ILA1 CD8^+^ T-cell clones. **(A)** Sequence details and binding affinities of native index peptide ILAKFLHWL of ubiquitous tumor-associated antigen human telomerase reverse transcriptase (hTERT_540–548_) with peptide variants IL**G**KFLHWL (3G), ILAKFLH**T**L (8T) and ILAKFLH**E**L (8E). Negative control peptide ELAGIGILTV (ELA). **(B)** Extracellular acidification rate (ECAR) analysis of ILA1 CD8^+^ T-cell clones stimulated with native index peptide, 3G, 8E, 8T and negative control ELA (all 10 µM). Final injection of 2-DG (100 mM). **(C)** Fold change of index peptide, 3G, 8T, 8E, and ELA. Fold ECAR change calculated with use of single measurement prior to peptide injection and single measurement after peptide injection. **(D)** Oxygen consumption rate (OCR) analysis of ILA1 stimulated with index peptide, 3G, 8E, 8T, and negative control ELA (all 10 µM) including **(E)** fold OCR change as calculated above. Data are representative of 6–10 technical repeats comprised of three independent experiments. Data expressed as mean + SEM; *p ≤ 0.05, ***p ≤ 0.001.

In order to investigate whether TCR affinity could tune T-cell metabolism, we used a panel of previously defined APLs that are recognized by the ILA1 TCR with a range of binding affinities (Figure 7A) (20). This is the first time T-cell metabolism has been investigated through natural ligands with altered affinity.

The hierarchy of ECAR fold change corresponded to the affinity of the ILA1 TCR for each APL, with the strongest affinity ligand (3G) generating the highest ECAR, and the lowest affinity ligand (8E) generating no ECAR increase (Figures 7B,C). This peptide stimulation initiated an increase in ECAR followed by a gradual decrease presumably as glucose in the original seeding media was utilized.

Analysis of OCR revealed that the rate of oxygen consumption was also dependent on the affinity of TCR–pHLA interaction (Figure 7D). This was reflected in the OCR fold change where index peptide and 8T APL had similar OCR fold changes and 3G APL had the largest OCR fold change (Figure 7E). Thus, we demonstrate, for the first time that TCR binding affinity governs the metabolic response to antigen, likely a critical step in determining T-cell effector functions.

## Discussion

Elucidating metabolic differences of leukocyte subsets and their changes over the life course of a cell is critical to our understanding of both basic immunology and perturbations with disease. Initially, the bioenergetics profile of quiescent, glucose starved CD4^+^ and CD8^+^ T-cells were compared to show that upon exposure to glucose T-cells exhibit no glycolytic reserve, although this was in a finite glucose scenario. While both populations were quickly at maximum glycolysis the CD4^+^ subset had significantly higher basal levels of glycolysis, which could be attributed to elevated glycolytic enzymes, specifically hexokinase isozyme II. These enzymes are important early in glycolysis for phosphorylating glucose to trap it inside the cell (34). Quiescent CD4^+^ T-cells also had higher oxygen consumption. Significantly higher levels of mitochondria, measured using MitoTracker, in CD4^+^ than CD8^+^ T-cells in all likelihood support this which is comparable to what has been reported previously in mice (11). Within the CD8^+^ T-cell population there was a MitoTracker^Hi^ and a MitoTracker^Lo^ subpopulation that could reflect mitochondrial biogenesis within the CD8^+^ T-cell population (44).

Like most other hematopoietic cell populations studied to date, human CD4^+^ and CD8^+^ T-cells undergo a “Warburg-like” switch to increased glycolytic metabolism upon activation; while accompanied by an increase in oxygen consumption, overall the balance shifts to favor glycolysis immediately upon cell stimulation. This would support ATP and biosynthetic intermediate production to fuel effector functions and we confirm a role for glucose consumption in cytokine production by both CD4^+^ and CD8^+^ T-cells. There were, however, some notable differences in the two T cell populations. CD8^+^ T-cellsexhibited a gradual decrease in glycolysis post-activation and showed greater dependency on mitochondrial metabolism for cytokine production. This gradual decrease in glycolysis by CD8^+^ T-cells only could reflect metabolites being directed to the mitochondria to support cytokine production and other effector functions. Differential expression of various glycolysis enzymes supports these differences in CD4^+^ and CD8^+^ T-cells and could allow CD4^+^ T-cells to maintain an elevated level of glycolysis. Here, we did not consider whether an alternative fuel switch, such as glutamine utilization as reported in murine CD4^+^ and CD8^+^ T-cells, occurs (11, 45). These differential effects of metabolic inhibition on cytokine production could provide targets for the control of inflammatory disease.

Immediate glycolytic switching was also shown using human CD4^+^ and CD8^+^ T-cell clones when activated with either the non-specific stimulus anti-CD3/CD28 or in an antigen-specific manner. ECAR and OCR were increased with natural peptide stimulation in comparison to anti-CD3/CD28. Further to this, we studied the ILA1 TCR, which binds with a range of affinities to altered peptides in order to determine whether TCR binding affinity could tune T-cell metabolism. These experiments demonstrated a clear relationship between TCR affinity and metabolic response, with the strongest ILA1 TCR affinity interaction for the 3G peptide inducing the greatest glycolytic change and the weakest, 8E, inducing the smallest glycolytic change. These findings were also consistent when oxygen consumption was investigated. This observation supports previous studies where strength of interaction between TCR and specific pHLA controls murine T-cell responses (46). Fine-tuning the ability of the HLA/peptide to promote post-TCR metabolic changes has implications for therapeutic manipulation of T-cells in cancer and for vaccination (47, 48).

The clinical importance and therapeutic potential of immunometabolism is emerging with modulation of glycolysis and mitochondrial respiration used increasingly to alter cell and disease phenotypes. This includes systemic lupus erythematosus where dual treatment of 2-DG and metformin normalizes CD4^+^ T-cell metabolism, and cancer where microRNA is being used to target glycolysis (49–51). There has also been considerable attention to the use of metformin alongside 2-DG and 6-diazo-5-oxo-l-norleucine (inhibitor of glutaminolysis) in a triple therapy strategy to prolong allograft survival by suppressing autologous T-cell rejection (42). Collectively, our findings illustrate differences in the metabolic activity of CD4^+^ and CD8^+^ T-cells and that TCR-pHLA affinity governs the underlying glycolytic switch and thus T-cell activation status. These findings aid our understanding of metabolically linked T-cell activation thresholds and could potentially improve vaccination strategies *via* the understanding of metabolic profiles during immune cell activation.

## Ethics Statement

All subjects gave written informed consent in accordance with the Declaration of Helsinki. Human blood samples were collected with informed written consent and ethical approval was obtained from Wales Research Ethics Committee 6 (13/WA/0190).

## Author Contributions

NJ performed the majority of experiments; JC, GD, AJS, SP, and SG performed experiments and provided intellectual discussion. AKS contributed reagents. NF, DC, NJ, and CT designed the experiments. NJ, DC, CT, and NF wrote the manuscript. All authors critically revised and approved the manuscript.

## Conflict of Interest Statement

The authors declare that the research was conducted in the absence of any commercial or financial relationships that could be construed as a potential conflict of interest.

## References

[1] Zloza A Al-Harthi L Multiple populations of T lymphocytes are distinguished by the level of CD4 and CD8 coexpression and require individual consideration. J Leukoc Biol (2006) 79:4–6. 10.1189/jlb.0805455 16380600

[2] Ma EH Bantug G Griss T Condotta S Johnson RM Samborska B Serine Is an essential metabolite for effector T cell expansion. Cell Metab (2017) 25:345–57. 10.1016/j.cmet.2016.12.011 28111214

[3] Ron-Harel N Santos D Ghergurovich JM Sage PT Reddy A Lovitch SB Mitochondrial biogenesis and proteome remodeling promote one-carbon metabolism for T cell activation. Cell Metab (2016) 24:104–17. 10.1016/j.cmet.2016.06.007 27411012 PMC5330619

[4] Mehta MM Weinberg SE Chandel NS. Mitochondrial control of immunity: beyond ATP. Nat Rev Immunol (2017) 17(10):608–20. 10.1038/nri.2017.66 28669986

[5] O’Neill LA Kishton RJ Rathmell J. A guide to immunometabolism for immunologists. Nat Rev Immunol (2016). 10.1038/nri.2016.70 27396447 PMC5001910

[6] O’Sullivan D van der Windt GJ Huang SC Curtis JD Chang CH Buck MD Memory CD8(+) T cells use cell-intrinsic lipolysis to support the metabolic programming necessary for development. Immunity (2014) 41:75–88. 10.1016/j.immuni.2014.06.005 25001241 PMC4120664

[7] Gubser PM Bantug GR Razik L Fischer M Dimeloe S Hoenger G Rapid effector function of memory CD8+ T cells requires an immediate-early glycolytic switch. Nat Immunol (2013) 14:1064–72. 10.1038/ni.2687 23955661

[8] Pollizzi KN Patel CH Sun IH Oh MH Waickman AT Wen J mTORC1 and mTORC2 selectively regulate CD8(+) T cell differentiation. J Clin Invest (2015) 125:2090–108. 10.1172/JCI77746 25893604 PMC4463194

[9] Cui G Staron MM Gray SM Ho PC Amezquita RA Wu J IL-7-induced glycerol transport and TAG synthesis promotes memory CD8+ T cell longevity. Cell (2015) 161:750–61. 10.1016/j.cell.2015.03.021 25957683 PMC4704440

[10] Henson SM Lanna A Riddell NE Franzese O Macaulay R Griffiths SJ p38 signaling inhibits mTORC1-independent autophagy in senescent human CD8(+) T cells. J Clin Invest (2014) 124:4004–16. 10.1172/JCI75051 25083993 PMC4151208

[11] Cao Y Rathmell JC Macintyre AN. Metabolic reprogramming towards aerobic glycolysis correlates with greater proliferative ability and resistance to metabolic inhibition in CD8 versus CD4 T cells. PLoS One (2014) 9:e104104. 10.1371/journal.pone.0104104 25090630 PMC4121309

[12] Phan AT Doedens AL Palazon A Tyrakis PA Cheung KP Johnson RS Constitutive glycolytic metabolism supports CD8+ T cell effector memory differentiation during viral infection. Immunity (2016) 45:1024–37. 10.1016/j.immuni.2016.10.017 27836431 PMC5130099

[13] Macintyre AN Gerriets VA Nichols AG Michalek RD Rudolph MC Deoliveira D The glucose transporter Glut1 is selectively essential for CD4 T cell activation and effector function. Cell Metab (2014) 20:61–72. 10.1016/j.cmet.2014.05.004 24930970 PMC4079750

[14] Cammann C Rath A Reichl U Lingel H Brunner-Weinzierl M Simeoni L Early changes in the metabolic profile of activated CD8(+) T cells. BMC Cell Biol (2016) 17:28. 10.1186/s12860-016-0104-x 27387758 PMC4937576

[15] Cretenet G Clerc I Matias M Loisel S Craveiro M Oburoglu L Cell surface Glut1 levels distinguish human CD4 and CD8 T lymphocyte subsets with distinct effector functions. Sci Rep (2016) 6:24129. 10.1038/srep24129 27067254 PMC4828702

[16] Verbist KC Guy CS Milasta S Liedmann S Kamiński MM Wang R Metabolic maintenance of cell asymmetry following division in activated T lymphocytes. Nature (2016) 532:389–93. 10.1038/nature17442 27064903 PMC4851250

[17] van der Windt GJ Everts B Chang CH Curtis JD Freitas TC Amiel E Mitochondrial respiratory capacity is a critical regulator of CD8+ T cell memory development. Immunity (2012) 36:68–78. 10.1016/j.immuni.2011.12.007 22206904 PMC3269311

[18] Wahl DR Byersdorfer CA Ferrara JL Opipari AW Jr Glick GD. Distinct metabolic programs in activated T cells: opportunities for selective immunomodulation. Immunol Rev (2012) 249:104–15. 10.1111/j.1600-065X.2012.01148.x 22889218 PMC3422770

[19] Shi LZ Wang R Huang G Vogel P Neale G Green DR HIF1alpha-dependent glycolytic pathway orchestrates a metabolic checkpoint for the differentiation of TH17 and Treg cells. J Exp Med (2011) 208:1367–76. 10.1084/jem.20110278 21708926 PMC3135370

[20] Laugel B van den Berg HA Gostick E Cole DK Wooldridge L Boulter J Different T cell receptor affinity thresholds and CD8 coreceptor dependence govern cytotoxic T lymphocyte activation and tetramer binding properties. J Biol Chem (2007) 282:23799–810. 10.1074/jbc.M700976200 17540778

[21] Huang J Zeng X Sigal N Lund PJ Su LF Huang H Detection, phenotyping, and quantification of antigen-specific T cells using a peptide-MHC dodecamer. Proc Natl Acad Sci U S A (2016) 113:E1890–7. 10.1073/pnas.1602488113 26979955 PMC4822615

[22] Tan MP Gerry AB Brewer JE Melchiori L Bridgeman JS Bennett AD T cell receptor binding affinity governs the functional profile of cancer-specific CD8+ T cells. Clin Exp Immunol (2015) 180:255–70. 10.1111/cei.12570 25496365 PMC4408161

[23] Tan MP Dolton GM Gerry AB Brewer JE Bennett AD Pumphrey NJ HLA class I-redirected anti-tumour CD4+ T-cells require a higher TCR binding affinity for optimal activity than CD8+ T-cells. Clin Exp Immunol (2017) 187(1):124–37. 10.1111/cei.12828 27324616 PMC5167017

[24] Acuto O Michel F CD28-mediated co-stimulation: a quantitative support for TCR signalling. Nat Rev Immunol (2003) 3:939–51. 10.1038/nri1248 14647476

[25] Fracchia KM Pai CY Walsh CM Modulation of T cell metabolism and function through calcium signaling. Front Immunol (2013) 4:324 10.3389/fimmu.2013.00324 24133495 PMC3795426

[26] Puleston DJ Villa M Pearce EL. Ancillary activity: beyond core metabolism in immune cells. Cell Metab (2017) 26:131–41. 10.1016/j.cmet.2017.06.019 28683280 PMC5546226

[27] Almeida L Lochner M Berod L Sparwasser T. Metabolic pathways in T cell activation and lineage differentiation. Semin Immunol (2016) 28:514–24. 10.1016/j.smim.2016.10.009 27825556

[28] Delmastro-Greenwood MM Piganelli JD. Changing the energy of an immune response. Am J Clin Exp Immunol (2013) 2:30–54. 23885324 PMC3714201

[29] Man K Miasari M Shi W Xin A Henstridge DC Preston S The transcription factor IRF4 is essential for TCR affinity-mediated metabolic programming and clonal expansion of T cells. Nat Immunol (2013) 14:1155–65. 10.1038/ni.2710 24056747

[30] Holland CJ Dolton G Scurr M Ladell K Schauenburg AJ Miners K Enhanced detection of antigen-specific CD4+ T cells using altered peptide flanking residue peptide-MHC class II MULTIMERS. J Immunol (2015) 195:5827–36. 10.4049/jimmunol.1402787 26553072 PMC4671089

[31] Purbhoo MA Li Y Sutton DH Brewer JE Gostick E Bossi G The HLA A*0201-restricted hTERT(540-548) peptide is not detected on tumor cells by a CTL clone or a high-affinity T-cell receptor. Mol Cancer Ther (2007) 6:2081–91. 10.1158/1535-7163.MCT-07-0092 17620437

[32] Theaker SM Rius C Greenshields-Watson A Lloyd A Trimby A Fuller A T-cell libraries allow simple parallel generation of multiple peptide-specific human T-cell clones. J Immunol Methods (2016) 430:43–50. 10.1016/j.jim.2016.01.014 26826277 PMC4783706

[33] Jones N Piasecka J Bryant AH Jones RH Skibinski DO Francis NJ Bioenergetic analysis of human peripheral blood mononuclear cells. Clin Exp Immunol (2015) 182:69–80. 10.1111/cei.12662 26032049 PMC4578510

[34] Marko AJ Miller RA Kelman A Frauwirth KA. Induction of glucose metabolism in stimulated T lymphocytes is regulated by mitogen-activated protein kinase signaling. PLoS One (2010) 5:e15425. 10.1371/journal.pone.0015425 21085672 PMC2978105

[35] Ganapathy-Kanniappan S Geschwind JF. Tumor glycolysis as a target for cancer therapy: progress and prospects. Mol Cancer (2013) 12:152. 10.1186/1476-4598-12-152 24298908 PMC4223729

[36] Shikhman AR Brinson DC Valbracht J Lotz MK. Cytokine regulation of facilitated glucose transport in human articular chondrocytes. J Immunol (2001) 167:7001–8. 10.4049/jimmunol.167.12.7001 11739520

[37] Zhao FQ Keating AF. Functional properties and genomics of glucose transporters. Curr Genomics (2007) 8:113–28. 10.2174/138920207780368187 18660845 PMC2435356

[38] Hresko RC Hruz PW. HIV protease inhibitors act as competitive inhibitors of the cytoplasmic glucose binding site of GLUTs with differing affinities for GLUT1 and GLUT4. PLoS One (2011) 6:e25237. 10.1371/journal.pone.0025237 21966466 PMC3179492

[39] Blagih J Coulombe F Vincent EE Dupuy F Galicia-Vázquez G Yurchenko E The energy sensor AMPK regulates T cell metabolic adaptation and effector responses in vivo. Immunity (2015) 42:41–54. 10.1016/j.immuni.2014.12.030 25607458

[40] Loreni F Thomas G Amaldi F. Transcription inhibitors stimulate translation of 5’ TOP mRNAs through activation of S6 kinase and the mTOR/FRAP signalling pathway. Eur J Biochem (2000) 267:6594–601. 10.1046/j.1432-1327.2000.01753.x 11054111

[41] Nguyen HD Chatterjee S Haarberg KM Wu Y Bastian D Heinrichs J Metabolic reprogramming of alloantigen-activated T cells after hematopoietic cell transplantation. J Clin Invest (2016) 126:1337–52. 10.1172/JCI82587 26950421 PMC4811142

[42] Lee CF Lo YC Cheng CH Furtmüller GJ Oh B Andrade-Oliveira V Preventing allograft rejection by targeting immune metabolism. Cell Rep (2015) 13:760–70. 10.1016/j.celrep.2015.09.036 26489460 PMC4626381

[43] Keppel MP Saucier N Mah AY Vogel TP Cooper MA Activation-specific metabolic requirements for NK cell IFN-gamma production. J Immunol (2015) 194:1954–62. 10.4049/jimmunol.1402099 25595780 PMC4323953

[44] Dimeloe S Mehling M Frick C Loeliger J Bantug GR Sauder U The immune-metabolic basis of effector memory CD4+ T cell function under hypoxic conditions. J Immunol (2016) 196:106–14. 10.4049/jimmunol.1501766 26621861

[45] Wang R Dillon CP Shi LZ Milasta S Carter R Finkelstein D The transcription factor Myc controls metabolic reprogramming upon T lymphocyte activation. Immunity (2011) 35:871–82. 10.1016/j.immuni.2011.09.021 22195744 PMC3248798

[46] Zehn D Lee SY Bevan MJ. Complete but curtailed T-cell response to very low-affinity antigen. Nature (2009) 458:211–4. 10.1038/nature07657 19182777 PMC2735344

[47] Buhrman JD Slansky JE Improving T cell responses to modified peptides in tumor vaccines. Immunol Res (2013) 55:34–47. 10.1007/s12026-012-8348-9 22936035 PMC3952016

[48] Comber JD Philip R. MHC class I antigen presentation and implications for developing a new generation of therapeutic vaccines. Ther Adv Vaccines (2014) 2:77–89. 10.1177/2051013614525375 24790732 PMC3991156

[49] Yin Y Choi SC Xu Z Perry DJ Seay H Croker BP Normalization of CD4+ T cell metabolism reverses lupus. Sci Transl Med (2015) 7:274ra218 10.1126/scitranslmed.aaa0835 PMC5292723 25673763

[50] Weinberg SE Chandel NS. Targeting mitochondria metabolism for cancer therapy. Nat Chem Biol (2015) 11:9–15. 10.1038/nchembio.1712 25517383 PMC4340667

[51] Zhao E Maj T Kryczek I Li W Wu K Zhao L Cancer mediates effector T cell dysfunction by targeting microRNAs and EZH2 via glycolysis restriction. Nat Immunol (2016) 17:95–103. 10.1038/ni.3313 26523864 PMC4684796

