# Supplementary material for: Metabolic Adaptation of Human CD4+ and CD8+ T-Cells to T-Cell Receptor-Mediated Stimulation
Source: Front Immunol. 2017 Nov 9;8:1516. doi: 10.3389/fimmu.2017.01516 (PMC5684100; doi:10.3389/fimmu.2017.01516)
Supplement: Supplementary file 1 [file data_sheet_1.docx]

Supplementary Material

**Metabolic adaptation of human CD4+ and CD8+ T cells to TCR mediated stimulation**

**Nicholas Jones^1^, James G. Cronin^1^, Garry Dolton^2^, Silvia Panetti^1^, Andrea J. Schauenburg^2^, Sarah A. E. Galloway^2^, Andrew K. Sewell^2^, David K. Cole^2^, Catherine A. Thornton^1,^ *†***

**and Nigel J. Francis^1,^ *†,* ^*^.**

*** Correspondence: Dr Nigel Francis,** Institute of Life Science, Swansea University Medical School, Swansea, SA2 8PP, UK.

****Corresponding Author: n.j.francis@swansea.ac.uk

**Supplementary Figure 1. (A)** Non-glycolytic acidification response of CD4+ and CD8+ T cells **(B)** Non-mitochondrial respiration and **(C)** proton leak of CD4+ and CD8+ T cell subsets Data from (A-C) five independent experiments are expressed as mean ± SEM.

**Supplementary Figure 2. (A)** Extracellular acidification rate and **(B)** oxygen consumption rate of CD4+ and CD8+ T cells upon stimulation with anti-CD3 (0.2 μg/mL) and anti-CD28 (20 μg/mL) or LEAF™ isotype controls; mouse IgG2a κ (MOPC-173) and mouse IgG1 κ (MOPC-21). A final injection of glycolysis competitive inhibitor, 2-deoxy-D-glucose (2-DG; 100 mM; Sigma) reduced ECAR indicating any increase was due to glycolytic activity. Data are from four independent experiments and expressed as mean + SEM.

**Supplementary Figure 3. (A)** Protein immunoblot representative of two matched donors, and respective densitometry showing **(B)** hexokinase I (HK), **(C)** hexokinase II, **(D)** phosphofructokinase (PFK), **(E)** glyceraldehyde-3-phosphate dehydrogenase (GAPDH), **(F)** pyruvate kinase (PKM2), and **(G)** lactate dehydrogenase (LDH) expression levels between 24 hour unstimulated and activated (anti-CD3; 2 μg/mL and anti-CD28; 20 μg/mL) CD4+ and CD8+ T cells. Full scan blots are shown in Supplementary Figure 11. Data are from four independent experiments with donor matched CD4+ and CD8+ T cells (A-D). Data expressed as mean + SEM; * p ≤ 0.05.

**Supplementary Figure 4. Effect of metabolic pathway inhibitors on cytokine outputs of CD4+ T cells.** CD4+ T cells were stimulated with anti-CD3 (2 μg/mL) and anti-CD28 (20 μg/mL) for 24 h in the presence of 2-deoxy-D-glucose (2-DG; 25 mM), oligomycin (1 μM) or vehicle (methanol) Cytokine outputs were also measured: **(A)** IL-4, **(B)** IL-10, **(C)** IL-13 and **(D)** IL-17, all expressed as pg/mL and are from three - five independent experiments. Data expressed as mean + SEM; * p ≤ 0.05, ** p ≤ 0.01.

**Supplementary Figure 5. Effect of metabolic pathway inhibitors on cytokine outputs of CD8+ T cells.** CD8+ T cells were stimulated with anti-CD3 (2 μg/mL) and anti-CD28 (20 μg/mL) for 24 h in the presence of 2-deoxy-D-glucose (2-DG; 25 mM), oligomycin (1 μM) or vehicle (methanol). Cytokine outputs were measured: **(A)** Granzyme B, and **(B)** MIP-1β. All data expressed as pg/mL and are representative of three independent experiments. Data expressed as mean + SEM; * p ≤ 0.05, ** p ≤ 0.01.

**Supplementary Figure 6. Peptide titration of T cell clones (A)** Extracellular acidification measured from CD4+ T cell clone DCD10 in response to its recognized index peptide, native Flu1 HA_306-318_, PKYVKQNTLKLAT (PKY) titrated at 10, 1 and 0.1 μM alongside a positive control anti-CD3 (0.2 μg/mL) and anti-CD28 antibodies (20 μg/mL) and **(B)** corresponding oxygen consumption was measured. **(C)** Extracellular acidification measured from CD8+ T cell clone ILA1 in response to its recognized index peptide ILAKFLHWL titrated at 10, 1, 0.1 and 0.01 μM alongside positive control anti-CD3 (0.2 μg/mL) and anti-CD28 antibodies (20 μg/mL) **(D)** corresponding oxygen consumption was measured. Data are from seven-ten biological repeats comprised of three independent experiments and expressed as mean + SEM.

**Supplementary Figure 7.** CD4+ T-cell clone DCD10 expresses HLA-DR and is capable of presenting a HLA-DR restricted peptide. **(A)** DCD10 stained with a monoclonal antibody (Ab) for HLA-DR (clone AC122, phycoerythrin (PE) conjugated, Miltenyi Biotec) confirming the ability of this clone to present HLA-DR1 restricted peptides. As expected, DCD10 also stained for CD3 (clone BW264/56, peridinin-chlorophyll-protein (PerCP) conjugated, Miltenyi Biotec), CD4 (clone M-T466, allophycocyanin (APC) conjugated, Miltenyi Biotec) and MHC class I (W6/32, APC conjugated, BioLegend), but not for CD8 (clone BW1350/80, APC conjugated, Miltenyi Biotec). **(B)** DCD10 was able to activate in response to cognate peptide (influenza hemagglutinin, residues 306-318, sequence PKYVKQNTLKLAT (PKY)) in the absence of an antigen presenting cell thus confirming functionally that DCD10 can present the PKY HLA-DR peptide. Very minimal activation of DCD10 was seen with an irrelevant HLA-DR peptide (5T4, residues 111-130, sequence FARRPPLAELAALNLSGSRL). Other controls for the assay included T-cells incubated alone to establish background activation, and incubation with phytohemaglutinin to act as a positive control. After overnight incubation supernatants were harvested and the amount of MIP-1β and IFNγ produced quantified by performing an ELISA (R&D systems). The concentration of peptide that is used in Figure 6 is indicated by the arrow.

**Supplementary Figure 8. Peptide titration of T cell clones (A)** Sequence details of native index peptide Flu1 HA_306-318_, PKYVKQNTLKLAT (PKY), Flu3 C-terminally modified variant of HA_306-318_ with Arginine at P11 PKYVKQNTLKLA**R** (11R) and negative control ILAKFLHWL (ILA). ECAR and OCR after stimulation of DCD10 CD4+ T cell clone with index, 11R and negative control ILA (all 10 μM). Final injection of 2-deoxy-D-glucose (100 mM) showing **(B and D)** summary data and **(C and E)** fold change of calculated with use of single measurement prior to peptide injection and single measurement after peptide injection. Data are representative of seven-ten biological repeats (A-E) with the exception of negative control ILA in (B-E) that was five repeats comprised of three-two independent experiments respectively. . Data expressed as mean + ** p ≤ 0.01, *** p ≤ 0.001..

**Supplementary Figure 9. Total protein immunoblots for freshly isolated CD4+ and CD8+ T-cells.** Full scan blots of Figure 2A**.** Protein immunoblot showing GLUT1, hexokinase I (HK), hexokinase II, phosphofructokinase (PFK), glyceraldehyde-3-phosphate dehydrogenase (GAPDH), pyruvate kinase (PKM2), lactate dehydrogenase (LDH) and actin expression levels between basal CD4+ and CD8+ T-cells. Black lines represent where blot has been cut horizontally. Lanes 1-4 CD4+ T-cells, lanes 5-8 CD8+ T-cells.

**Supplementary Figure 10. Total protein immunoblots for 24 hour unstimulated and activated CD4+ and CD8+ T-cells.** Full scan blots of Figure 4A. Protein immunoblot representative of one matched donor showing GLUT1, ribosomal proteins; phospho-S6, total S6 and actin expression levels between 24 hour unstimulated and activated (anti-CD3; 2 μg/mL and anti-CD28; 20 μg/mL) CD4+ and CD8+ T cells. Black lines represent where blot has been cut horizontally. Lane 1 – CD4+ unstimulated, lane 2 – CD8+ unstimulated, lane 3 – CD4+ activated and lane 4 – CD8+ activated.

**Supplementary Figure 11. Total glycolytic enzyme immunoblots for 24 hour unstimulated and activated CD4+ and CD8+ T-cells.** Full scan blots shown of Supplementary Figure 3A. Protein immunoblot representative of two matched donors showing hexokinase I (HK), hexokinase II, phosphofructokinase (PFK), glyceraldehyde-3-phosphate dehydrogenase (GAPDH), pyruvate kinase (PKM2), lactate dehydrogenase (LDH) and actin expression levels between 24 hour unstimulated and activated (anti-CD3; 2 μg/mL and anti-CD28; 20 μg/mL) CD4+ and CD8+ T cells. Black lines represent where blot has been cut horizontally. Data are donor matched CD4+ and CD8+ T cells.
